# Home-Prepared Meal Consumption Is Associated with Healthy Food Choices in Pregnant Women Followed Up by Primary Health Care

**DOI:** 10.3390/ijerph192416557

**Published:** 2022-12-09

**Authors:** Mariana S. Gondin, Henrique P. Aguiar, Érika S. O. Patriota, Walkyria O. Paula, Nathalia Pizato, Sylvia C. C. Franceschini, Vivian S. S. Gonçalves

**Affiliations:** 1 Department of Nutrition, University of Brasilia, Campus Universitário Darcy Ribeiro S/N, Asa Norte, Brasilia 70910-900, DF, Brazil; 2 Graduate Program in Public Health, Faculty of Health Sciences, University of Brasilia, Campus Universitário Darcy Ribeiro S/N, Asa Norte, Brasilia 70910-900, DF, Brazil; 3 Graduate Program in Human Nutrition, Faculty of Health Sciences, University of Brasilia, Campus Universitário Darcy Ribeiro S/N, Asa Norte, Brasilia 70910-900, DF, Brazil; 4 Graduate Program in Nutrition Sciences, Federal University of Viçosa—UFV, Edifício Centro de Ciências Biológicas II, Campus Universitário, S/N, Viçosa 36570-900, MG, Brazil

**Keywords:** home-prepared meals, ultra-processed foods, primary health care, pregnancy weight gain, pregnant women

## Abstract

The act of preparing food, especially at home, may be related to improvement in healthy eating patterns. This study analyzed the association between home-prepared meals consumption and the food markers consumption, and weight gain in pregnant women followed up in Primary Health Care in the Federal District (DF), Brazil. This is a cross-sectional study, conducted with pregnant women of all gestational ages. The characteristics of meals preparation and intake, as well as the consumption of food markers, were evaluated through structured questionnaires. Gestational weight gain was evaluated based on data recorded in the pregnant woman’s booklet. Variables related to pregnancy, health, lifestyle, and socioeconomic status were analyzed as covariates. A total of 233 pregnant women were included in this study, with a mean age of 28.50 (SD = 6.32) years. Inadequate gestational weight gain was found in 46% of pregnant women. Consumption of soft drinks was 49% lower in pregnant women who prepared all meals at home. Eating home-prepared meals was inversely associated with a high score for unhealthy foods. Home-prepared meals consumption could be an effective health promotion strategy in Primary Health Care, helping to increase the chances of vegetable consumption, and decrease the consumption of soft drinks.

## 1. Introduction

Pregnancy is a period characterized by several physical, psychological, and biological changes. This stage triggers bodily processes to achieve the mother’s and child’s nutrition and development needs. The adequate quality of pregnant woman’s diet and her consequent nutritional status play a major role in fetal growth and pregnancy conditions and outcomes [1].

An important predictor of adverse outcomes during and after pregnancy is the women’s pre-gestational nutritional status, given the nutritional requirements demanded by the body and the fetus [2]. According to data from the Food and Nutrition Surveillance System, SISVAN, of the 11,209,406 Brazilian women monitored in 2019, 63.2% were classified as overweight according to body mass index. This means that about 7 million adult women followed up in Primary Health Care (PHC) were classified as overweight or obese [3]. 

The gestational weight gain is related to the women’s pre-gestational weight and is modulated by the increase in nutritional need-energy demand, macro- and micronutrients-provided by fetal growth, eating behavior, and hormonal changes. Excess weight gain during pregnancy can lead to several adverse outcomes [4], including the possibility of fetal macrosomia, gestational diabetes, large babies at birth, and an increased chance of cesarean deliveries [5].

During pregnancy, eating patterns may change from a precedent scenario of emotional and social context habits and attitudes combined with the presence of new beliefs, desires, and convictions that commonly arise during this period. Adequate food consumption provides the necessary resources for an environment conducive during pregnancy [4]. Over the past few decades, the participation of women in the workplace has increased worldwide [6], creating the habit of having meals outside of home. Considering the recent changes in dietary patterns, especially the increase in ultra-processed foods consumption, the pregnancy group should receive special attention due to the complications associated with malnutrition [7]. 

Ultra-processed foods are classified as high-energy-density foods with low nutritional quality, usually high in sugar, sodium, and fat. They promote excessive weight gain, and interfere with the consumption of healthier foods, as a matter of choice and opportunity [8]. In a literature review evaluating the food consumption of pregnant women carried out by Bueno et al. (2016), the inadequacy of vegetable and fruit consumption and a low fiber intake were identified, in disagreement with the recommendations of the World Health Organization (WHO) [9]. In 2022, a meta-analysis of cohort studies from different countries showed an association between the consumption of diets rich in ultra-processed foods with gestational diabetes and preeclampsia [10].

Considering the demand for practical and fast food, ultra-processed foods offer convenience to the consumer, while reducing the need for cooking and the skills involved in this process. The act of preparing food, especially at home, may be related to a higher quality of the diet, as well as an improvement in healthy eating patterns. Eating outside the home environment can be subject to choices with low micronutrients, portions of different sizes, and higher caloric value, which is related to the difficulty of food-related self-regulation [11].

Considering that higher consumption of ultra-processed foods contributes to excessive weight gain, the place where meals are prepared impacts the quality of food, and weight gain above the recommendations during pregnancy can negatively affect health, the investigation into the relationship between these issues can be a useful instrument to build a better understanding of protective or risk factors in order to achieve adequate food intake during pregnancy. 

Thus, the objective of this study was to analyze the relationship between home-prepared meals and the healthy and unhealthy patterns of food markers consumption and weight gain in pregnant women followed up at PHC in Federal District (FD).

## 2. Materials and Methods

### 2.1. Study Design

This is an epidemiological, observational, cross-sectional study, part of the Multicenter Study of Iodine Deficiency in the Maternal and Infant Population (EMDI-Brazil).

### 2.2. Study Population

The sample studied consisted of pregnant women followed up at PHC in FD. The data were collected between August 2019 and September 2021, during periods not affected by the COVID-19 pandemic quarantine restriction.

A simple random sample calculation was considered and performed using the StatCalc tool of the EpiInfo Software (Center for Disease Control and Prevention, USA). The sample size calculation took into account the average monthly prenatal care appointments at PHC in 2016, as a proxy for the number of pregnant women monitored by PHC in FD (*n* = 18,877; data reported by the DF State Department of Health), and the prevalence of the indicator “consumption of ultra-processed foods the day before” among Brazilian pregnant women monitored by PHC (81.5%), in the same year, from SISVAN [12]. The acceptable error of 5.5% and the 95% confidence interval (95%CI) were considered. Thus, the minimum number of pregnant women to be evaluated was defined as 190; 20% was added to the estimated number, anticipating possible losses, thus the sample was estimated at 228 pregnant women.

Ten PHC units were included by simple random selection, according to proximity proportional probability to the central region of the administrative organization and the highest monthly average prenatal care performed in 2016 (data reported by the State Department of Health of DF). In the PHC units, pregnant women were approached after medical prenatal appointments. Those who showed interest in entering the study and met the eligibility criteria were included in the study.

### 2.3. Eligibility Criteria

Women of all gestational ages (first, second, and third trimesters), residents in urban areas of FD, aged 18 years or older, were considered eligible for evaluation. Those diagnosed with hypothyroidism and pregnant women with a history of thyroid disease and/or surgery were not included, due to possible interference with the outcomes investigated in EMDI-Brazil.

### 2.4. Variables

All study variables were obtained through the application of structured questionnaires by trained researchers.

#### 2.4.1. Dependent Variables

The following were considered dependent variables: (a) consumption of food markers of healthy and unhealthy patterns-assessed individually and by score; and (b) gestational weight gain (GWG) up to the time of the interview.

The consumption of food markers was investigated using the Surveillance System for Risk and Protection Factors for Chronic Diseases by Telephone Survey (Vigitel) instrument, with adaptations [13]. This instrument consists of 23 questions whose answers (“yes” or “no”) are based on food markers consumption on the previous day. Each question pointed to a set of foods divided into two categories: in natura and minimally processed foods (13 sets) and ultra-processed foods (10 sets). The set of foods answered as “yes” was considered a marker of its presence in the diet of pregnant women.

In addition, a score of healthy and unhealthy foods was calculated, in which each food group consumed was assigned 1 point, with a scale of 0–13 for healthy and 0–10 for unhealthy. The 90th percentile of the distribution of scores in the evaluated population was used as a cut-off point to identify those who consumed the most healthy or unhealthy foods.

To assess the GWG, pre-gestational weight (PGW), date of last menstrual period (LMP), current weight (BP), age, and height were collected from the notes on the last prenatal appointment in the pregnant woman’s booklet. The pregnant woman’s booklet is an instrument that records all medical procedures and examinations performed during the prenatal period, as well as the progress of the pregnancy, facilitating the flow of information between healthcare professionals. The booklet documents all prenatal data, including medical appointments, exams, vaccines, anthropometric measures, and any other relevant information for prenatal follow-up. 

The information was used to classify gestational weight gain; as proposed by Kac et al. (2021), Brazilian women 10 weeks into gestation and those less than 10 weeks were not considered in the GWG analysis [14]. For this study, it was considered below the expected GWG <25th percentile; according to the expected GWG between the 25th percentile and the 75th percentile; and above expected-or excessive-GWG percentiles >75.

#### 2.4.2. Independent Variables

In the study, pregnant women were asked about the type of food they ate (home-prepared or not) and how often they used to have home-prepared food during a normal week. The independent variable and its response categories were then obtained and analyzed: consumption of food prepared at home at all meals, 7 days a week (breakfast, morning snack, lunch, afternoon snack, dinner, and supper)—yes or no.

#### 2.4.3. Covariates

The other variables used to characterize the pregnant women evaluated and categorized were as follows: skin color (white, brown, black, or Asian); education, considering complete or incomplete education (no education, elementary school, high school, or higher education); paid work in the previous month (yes or no); household income in the previous month (less than USD 92.00; between USD 92.00 and USD 185.00; between USD 185.01 and USD 551.00; above USD 551.00 or not informed); assisted by Brazilian social Bolsa Familia Program (yes or no); cohabitation with a spouse or partner (yes or no); and self-recognition as head of the family (yes or no).

Variables related to pregnancy, health, and lifestyle of pregnant women were also obtained: gestational trimester (first, second or third); the number of previous pregnancies (none, between 1 and 3, between 4 and 6, or more than 6); medical diagnosis of arterial hypertension before pregnancy (yes or no); current use of micronutrient supplement (yes or no); current use of cigarettes (yes or no); current consumption of alcoholic beverages (yes or no); and pre-gestational nutritional status by the Body Mass Index—BMI (low weight, normal weight, overweight and obesity) [15].

### 2.5. Data Analysis

For data analysis, measures of central tendency were expressed as mean and standard deviation (SD), when it was a continuous variable. To calculate the sample frequency related to categorical variables, the prevalence within the sample was estimated with their respective 95% confidence intervals (95% CI). The association between the variables of interest was investigated through robust Poisson Regression, with an estimation of crude and adjusted Prevalence Ratio (PR) and its 95%CI. For the adjusted PR, the variables that had gross PR with *p* < 0.20 were selected, and the following control variables were used: schooling, household income, and paid work in the previous month. The statistical software Stata 17 (StataCorp, College Station, TX, USA) was used for the analysis, and the significance of *p* < 0.05 was considered statistically significant.

## 3. Results

Data from 233 pregnant women followed up at 10 PHC units in DF were included. The mean age was 28.50 years old (SD = 6.32). Self-reported brown skin color was the most prevalent among the participants with incomplete or complete high school education. More than half of the pregnant women reported not having paid work in the previous month, and 12% were assisted by the Brazilian social cash transfer program (Bolsa Familia Program). Most of them lived with a partner, and about 34% recognized themselves as head of the family (Table 1).

The information about the participant’s nutritional status before pregnancy, as well as gestational weight gain data, were also evaluated. Among pregnant women, 44% were in the third trimester of pregnancy and 55% of them had already been pregnant at least once before. Previous arterial hypertension diagnosis and micronutrient supplementation during pregnancy were not reported by most of pregnant women. Smoking and alcohol drinking were reported by a few participants. According to pre-gestational BMI, about 47% of the women were eutrophic. Regarding gestational weight gain, 46% presented inadequate gain (Table 2).

Among healthy food markers, the highest consumption frequencies were observed in the rice group, followed by the meat and pulses groups. In contrast, dark green vegetables, eggs, and fruits had the lowest frequencies (Table 3).

The weekly average of home-prepared meals consumption was 5.75 days (SD = 2.47) while eating outside home was 0.79 days (SD = 1.86), and 76.1%; 95% CI (70.08–81.24) pregnant women reported consuming all meals prepared at home.

Regarding unhealthy food markers, the highest consumption frequencies among pregnant women were margarine and processed sauces; soft drinks; and processed meat products. In contrast, instant noodles, packet soup, frozen lasagna, or other frozen ready-to-eat dishes had the lowest frequencies. 

The results of the association between the consumption of healthy and unhealthy food markers, gestational weight gain, and consumption of home-prepared meal are presented in Table 4. Pregnant women who prepared all of their meals at home consumed 84% more foods from the pumpkin group, while soft drink consumption was 49% lower.

The healthy food markers score ranged from 1 to 13 points and the average was 6.55 points (SD = 2.32) and the unhealthy food markers score ranged from 0 to 10 points and the 2.54 points (SD = 1.68). Consuming home-prepared meals was inversely associated with a higher score of unhealthy foods (>90th percentile) (Table 5).

## 4. Discussion

This study highlights the relationship between home-prepared meals, healthy and unhealthy food markers consumption, and gestational weight gain in pregnant women followed up in PHC. There is growing evidence that the place where meals are prepared can influence the quality of the diet and promote better health outcomes. A study including 35,084 adults showed that frequent consumption of meals prepared away from home is significantly associated with an increased risk of all-cause mortality [16].

The main results found in our study showed that having home-prepared meals was associated with higher food markers of a healthy dietary pattern consumption—such as the set consisting of pumpkin, carrots, sweet potatoes, or okra/caruru—and a lower intake of soft drinks, one of the unhealthy diet markers. A higher prevalence of unhealthy foods consumption score below the 90th percentile was observed in pregnant women that consumed all meals prepared at home. In general, a higher consumption of healthy food markers, especially fruits and vegetables, is observed when compared to unhealthy food markers during pregnancy [17], highlighting that the place where meals are prepared may be associated with the quality of food.

A study conducted between 2015 and 2016 with data from 150 families in the United States found that having home-prepared meals promoted higher chances of fruit and vegetable consumption [18]. The adequate consumption of these food groups is especially important during pregnancy because they provide important nutrients necessary for the development of the fetus and the maintenance of the health of the pregnant woman [19]. Yellow-orange vegetables—such as those present in the group of pumpkin, carrots, sweet potatoes, or okra/pigweed—are rich in β-carotene, a nutrient with important antioxidant capacity and a precursor of vitamin A, which is essential for embryonic development [20]. In addition, the consumption of a healthy dietary pattern rich in vegetables, fiber, and micronutrients is associated with a reduction in adverse maternal and child outcomes [21]. Therefore, the act of cooking foods at home becomes relevant as a strategy to include these foods in the maternal diet. Such a recommendation is also claimed by the Dietary Guideline for the Brazilian Population (2014), which strongly encourages the development, practicing, and sharing of culinary skills [22]. 

Another finding of this study showed that consuming home-prepared meals was inversely associated with a higher score of unhealthy foods (mainly ultra-processed foods), which reinforces the importance of preparing food at home as a strategy to reduce ultra-processed food consumption. Moreover, the consumption of home-prepared meals every day of the week decreased the prevalence of soft drink intake. The unhealthy food score distribution in this sample was similar to that observed in the general adult population, which also ranged from 0 to 13, with a higher frequency of scores above 5 [23].

Soft drink intake is an unhealthy food marker, and it is linked to a diet rich in ultra-processed foods [13]. These ultra-processed beverages generally have a high amount of sugar, as well as high energy density. A study conducted with data from 2578 low-income American adults in 2016 aimed to examine the associations between home-prepared meal, weekly consumption of fast foods, and their relationship with calories intake. The results showed that the consumption of home-cooked meals was associated with a lower intake of soft drinks, corroborating the results found in the present study [24].

Epifanio et al. (2020) examined the records obtained by the Brazilian Vigitel telephone survey conducted between 2007 and 2014 and observed a reduction in the trend of soft drink consumption in Brazilian adult population. Despite this, soft drink consumption is still high, with an average intake of one glass of soda per day, representing about 30 g of sugar [25]. High sugary drinks consumption is related to negative health outcomes, such as deterioration of oral health, a direct association with risk of bone fractures, increased insulin resistance, and hypertriglyceridemia in adult population [26,27]. During pregnancy, the consumption of soft drinks and other sugary drinks was also associated with maternal hypertensive disorders and gestational diabetes [10].

It is also noteworthy to point out that among the pregnant women evaluated in our study, we observed 49% of gestational weight gain inadequacy, either insufficient or excessive. Gestational weight gain is also influenced by maternal food consumption. A cohort study of pregnant women with obesity showed that higher intake of ultra-processed foods was predictive of excessive gestational weight gain [28]. In the results of this study, the home-prepared meals were associated with higher diet quality, which was influenced by the lower consumption of ultra-processed foods, a risk factor for excessive weight gain.

The consumption of ultra-processed foods is associated with lower diet quality, since it promotes higher intake of sugar, sodium, and saturated fats and a reduction in the consumption of fiber, vitamins, and micronutrients [29]. A cohort study conducted with 259 Brazilian pregnant women between 2012 and 2014 found a positive association between ultra-processed foods consumption in the third trimester of pregnancy and an average gain of 4.17 g per week [30]. A recent systematic review reported that dietary patterns rich in ultra-processed foods were associated with greater gestational weight gain, which contributes to negative outcomes for the fetus, and increases the risk of venous thromboembolism, depression, and difficulties in breastfeeding [31].

Among the healthy food markers analyzed in this study, the consumption of cereals, meats, and pulses was more prevalent, while the consumption of vegetable in general had a low frequency of consumption. Regarding unhealthy food markers, industrialized sauces, soft drinks, and processed meat products were the most frequent items consumed. These results are similar to those found by the Brazilian Consumer Expenditure Survey (2017–2018) which showed that rice (cereal group) and beans (legume group) were among the most consumed foods by Brazilian families, whereas the vegetable group was among the foods with the lowest consumption [32]. These findings corroborate Camargo et al. (2012), who conducted a cross-sectional study with pregnant women attended at PHC unit and observed higher prevalence of consumption of foods such as rice and beans in the evaluated group [33].

Although it was not the main objective of this study, it is noteworthy to point another finding among the evaluated participants. Alcohol consumption and smoking were reported by some pregnant women. Alcoholism and smoking in pregnant women are associated with increased risks of premature birth, low birth weight, and with negative impacts on children’s health, being strongly contraindicated to this group [34].

This study has some limitations that need to be considered when interpreting the results. The cross-sectional design does not allow the establishment of causality between the analyzed variables. Furthermore, this design limits to generalizing the results, considering the heterogeneity of the physiological, and emotional status in all three different trimesters. Another point to be noted is that the self-reported food consumption investigation might be affected by memory bias. In addition, since it is a recently developed instrument, there is no standard score for healthy and unhealthy foods for pregnant women. Therefore, reference groups are not available for comparison. Despite the limitations, it is worth highlighting the strengths of this study. To the authors’ knowledge, this is the first study with Brazilian pregnant women that investigated the influence of the place where meals are prepared on healthy or unhealthy food markers choices and gestational weight gain. Data collection was carried out with strong methodological rigor by trained researchers, and data analysis was conducted using robust methods.

## 5. Conclusions

The results of the present study indicate that having home-prepared food during pregnancy was associated with higher consumption of vegetables, such as pumpkin, carrots, sweet potatoes or okra/caruru, and a lower intake of soft drinks. It is worth mentioning the importance of valuing dietary guidelines, in order to promote the importance of cooking meals at home, especially during pregnancy period. These findings may support future investigations on the subject, contributing to the guidance of nutritional guidelines during pregnancy, a period in which food plays a crucial role in promoting favorable outcomes, both for the mother and the child.

## Figures and Tables

**Table 1:** Social, economic, and demographic characteristics of pregnant women followed up in Primary Health Care in the Federal District, 2019–2021.

Characteristics		Prevalence	
	*n*	%	CI95%
Skin color/ethnicity			
White	45	19.31	14.76–24.86
Brown	137	58.80	52.39–64.93
Black	36	15.46	11.37–20.65
Asian	8	3.43	1.75–6.63
Not reported	7	3.00	1.56–6.07
Schooling level ^a^			
No Schooling	1	0.43	0.08–2.39
Elementary School	43	18.45	14.00–23.93
High School	128	54.94	48.52–61.19
Higher Education	55	23.60	18.61–29.46
Not reported	6	2.58	1.19–5.50
Paid work in the previous month			
No	105	45.06	38.81–51.48
Yes	122	52.36	45.96–58.68
Not reported	6	2.58	1.19–5.50
Household income in the previous month			
Less than USD 92.00	13	5.58	3.29–9.31
Between USD 92.00 and USD 185.00	21	9.01	5.97–13.38
Between USD 185.01 and USD 551.00	100	42.92	36.73–49.34
Above USD 551.00	59	25.32	20.17–31.27
Not Reported	40	17.17	12.87–22.53
Assisted by Bolsa Família Program			
No	198	84.98	79.83–89.00
Yes	28	12.02	8.45–16.82
Not reported	7	3.00	1.56–6.07
Lives with partner			
No	50	21.46	16.67–27.17
Yes	177	75.96	70.09–81.00
Not reported	6	2.58	1.19–5.50
Self-recognition as head of the family			
No	148	63.51	57.17–69.43
Yes	79	33.91	28.13–40.22
Not reported	6	2.58	1.19–5.50

Legend: CI95% = Confidence Interval 95%; ^a^ complete or incomplete level; *n* = 233.

**Table 2:** Characteristics related to pregnancy, health and lifestyle of pregnant women followed up in Primary Health Care in the Federal District, 2019–2021.

Characteristics	Prevalence		
	*n*	%	CI95%
Gestational Trimester			
First	35	15.02	11.00–20.17
Second	91	39.06	33.02–45.45
Third	103	44.20	37.97–50.63
Not reported	4	1.72	0.67–4.33
Number of previous pregnancies			
None	90	38.62	32.56–45.06
Between 1 to 3	129	55.36	48.89–61.65
Between 4 to 6	13	5.60	3.25–9.39
More than 6	1	0.42	0.05–3.01
Arterial Hypertension prior to pregnancy ^a^			
Yes	11	4.72	2.66–8.52
No	217	93.13	89.14–95.73
Not reported	5	2.15	0.92–4.92
Current use of micronutrient supplement			
Yes	192	82.40	77.00–86.76
No	37	15.88	11.75–21.12
Not reported	4	1.72	0.67–4.33
Smoking			
Yes	9	3.86	2.05–7.18
No	218	93.56	89.65–96.06
Not reported	6	2.58	1.19–5.50
Current intake of alcoholic beverage			
Yes	27	11.59	8.09–16.33
No	197	84.55	79.35–88.63
Not reported	9	3.86	2.05–7.18
Pre–gestational nutritional status ^b^			
Underweight	9	3.86	2.05–7.18
Normal weight	109	46.79	40.48–53.19
Overweight	59	25.32	20.17–31.27
Obesity	33	14.16	10.27–19.22
Not reported	23	9.87	6.67–14.38
Gestational Weight Gain ^c^			
<P25	62	26.61	21.35–32.63
P25–P75	101	43.35	37.14–49.77
>P75	46	19.74	15.14–25.33
Not reported	24	10.29	7,02–14.87

Legend: CI95% = Confidence Interval 95%; ^a^ Previous diagnosis by a doctor; ^b^ Body Mass Index; ^c^ Kac and Carrilho et al. (2021) classification used to pregnant women over 10 gestational weeks; *n* = 233; P = percentile.

**Table 3:** Healthy and unhealthy eating markers consumption of pregnant women followed up in Primary Health Care in the Federal District, 2019–2021.

Characteristics	All Pregnant Women (*n* = 226)		Pregnant Women Who Consume All Meals Prepared at Home (*n* = 172)		Pregnant Women Who Do Not Consume All Meals Prepared at Home (*n* = 54)	
	%	CI95%	%	CI95%	%	CI95%
Healthy eating markers						
Rice, pasta, polenta, couscous or corn	95.13	91.40–97.29	95.32	90.88–97.65	94.33	83.48–98.21
Potato, cassava or yam	38.05	31.92–44.58	40.35	33.21–47.92	30.18	19.17–44.07
Beans, peas, lentils or chickpeas	73.00	66.81–78.42	73.10	65.90–79.25	71.69	57.87–82.36
Beef, pork, chicken or fish	89.38	84.61–92.79	88.89	83.18–92.82	90.56	78.89–96.10
Fried, boiled or scrambled egg	25.66	20.36–31.79	27.49	21.27–34.70	16.98	8.93–29.88
Kale, broccoli, watercress or other dark green leafy vegetables	19.46	14.79–25.18	20.47	15.04–27.22	13.20	6.31–25.56
Lettuce, Swiss chard, cabbage or other light green leafy vegetables	37.16	31.08–43.68	40.35	33.21–47.92	26.41	16.11–40.14
Pumpkin, carrots, sweet potatoes or okra/caruru	34.95	28.98–41.43	38.60	31.55–46.15	20.75	11.71–34.07
Tomato, cucumber, zucchini, eggplant or any other vegetable	55.30	48.73–61.70	53.80	46.24–61.18	58.49	44.60–71.14
Papaya, mango, yellow melon or pequi	28.31	22.79–34.57	29.82	23.40–37.15	24.52	14.62–38.14
Orange, banana, apple, pineapple or any other fruit	60.17	53.62–66.38	61.99	54.43–68.99	52.83	39.20–66.04
Milk	58.84	52.28–65.11	61.99	54.43–68.99	50.94	37.43–64.31
Any kind of cheese	34.51	28.57–40.98	31.58	25.01–38.97	41.50	28.85–55.39
Unhealthy food markers						
Soft drinks	29.64	24.02–35.95	25.15	19.17–32.23	45.28	32.23–59.01
Bottled or canned juice, fruit drink prepared from powdered mix	22.56	17.55–28.50	22.80	17.09–29.74	20.75	11.71–34.07
Chocolate drink or flavored yogurt	23.89	18.75–29.91	21.63	16.06–28.48	28.30	17.63–42.12
Salty snacks or crackers/cookies	21.68	16.76–27.56	22.22	16.57–29.11	20.75	11.71–34.07
Sweet snacks or cookies or processed cake/cupcake	21.23	16.36–27.08	19.89	14.53–26.59	26.41	16.11–40.14
Ice cream, chocolate, gelatin, flan or other industrialized dessert	22.12	17.16–28.03	20.47	15.04–27.22	28.30	17.63–42.12
Sausage, Bologna, salami or ham	26.66	21.27–32.86	24.70	18.76–31.79	32.07	20.73–46.01
Buns, rolls or any type of packaged breads	25.22	19.96–31.32	24.56	18.65–31.61	28.30	17.63–42.12
Margarine, mayonnaise, ketchup or other industrialized sauces	49.11	42.61–55.64	46.20	38.81–53.75	58.49	44.60–71.14
Instant noodles, packet soup, frozen lasagna or other frozen ready-to-eat dishes	7.07	4.37–11.26	7.60	4.44–12.69	5.66	1.78–16.51

Legend: CI95% = Confidence Interval 95%; *n* = 226.

**Table 4:** Association between consumption on the previous day of food markers of dietary patterns, gestational weight gain, and consumption of home-prepared meals of pregnant women in the Federal District, EMDI-Brazil, 2019–2021.

Characteristics	Consumption of All Meals Prepared at Home			
	Crude PR	CI95%	Adjusted PR	CI95%
Healthy eating markers				
Rice, pasta, polenta, couscous or corn	1.01	0.93–1.08	-	
Potato, cassava or yam	1.33	0.85–2.09	-	
Beans, peas, lentils or chickpeas	1.01	0.84–1.23	-	
Beef, pork, chicken or fish	0.98	0.88–1.08	-	
Fried, boiled or scrambled egg	1.61	0.84–3.08	1.56	0.83–2.95
Kale, broccoli, watercress or other dark green leafy vegetables	1.54	0.73–3.28	1.48	0.70–3.12
Lettuce, Swiss chard, cabbage or other light green leafy vegetables	1.52	0.93–2.48	1.49	0.91–2.43
Pumpkin, carrots, sweet potatoes or okra/caruru	1.85	1.06–3.25	1.84	1.05–3.23 *
Tomato, cucumber, zucchini, eggplant or any other vegetable	0.91	0.70–1.20	-	
Papaya, mango, yellow melon or pequi	1.21	0.71–2.05	-	
Orange, banana, apple, pineapple or any other fruit	1.17	0.88–1.55	1.15	0.86–1.52
Milk	1.21	0.91–1.62	1.25	0.94–1.67
Any kind of cheese	0.76	0.51–1.12	0.75	0.51–1.10
Unhealthy food markers				
Soft drinks	0.55	0.37–0.82	0.49	0.33–0.74 *
Bottled or canned juice, fruit drink prepared from powdered mix	1.09	0.60–1.99	-	-
Chocolate drink or flavored yogurt	0.76	0.45–1.28	-	-
Salty snacks or crackers/cookies	1.07	0.58–1.94	-	-
Sweet snacks or cookies or processed cake/cupcake	0.75	0.43–1.29	-	-
Ice cream, chocolate, gelatin, flan or other industrialized dessert	0.72	0.42–1.21	0.66	0.39–1.11
Sausage, Bologna, salami or ham	0.77	0.48–1.23	0.82	0.51–1.32
Buns, rolls or any type of packaged breads	0.86	0.52–1.43	-	-
Margarine, mayonnaise, ketchup or other industrialized sauces	0.78	0.59–1.04	0.77	0.58–1.02
Instant noodles, packet soup, frozen lasagna or other frozen ready-to-eat dishes	1.34	0.39–4.54	-	-
Gestational Weight Gain				
Adequate	1.23	0.84–1.79	1.29	0.89–1.87

Legend: *n* = 226; pregnant women with available gestational weight gain data = 202; PR = Prevalence Ratio; CI95% = Confidence Interval 95%; * *p* < 0.05; Variables with *p* < 0.20 in the crude analysis were selected for adjusted analysis. The following control variables were used in the adjusted analysis: schooling, household income, and paid work in the previous month; *n* = 226.

**Table 5:** Association between healthy and unhealthy scores for consumption of foods markers of dietary patterns and consumption of home-prepared meals by pregnant women in the Federal District, EMDI-Brazil, 2019–2021.

Scores	Consumption of All Meals Prepared at Home			
	Crude PR	CI95%	Adjusted PR	CI95%
Healthy	2.47	0.77–7.93	2.29	0.70–7.43
Unhealthy	0.48	0.24–0.96	0.41	0.21–0.80 *

Legend: *n* = 226; PR = Prevalence Ratio; CI95% = Confidence Interval 95%; * *p* < 0.05; Variables with *p* < 0.20 in the crude analysis were selected for adjusted analysis. The scores considered the 90th percentile of the sample as the cutoff point (*p* 90 of the healthy marker score = 10; unhealthy marker score = 5 points). The following control variables were used in the adjusted analysis: schooling, household income, and paid work in the previous month; *n* = 226.

## Data Availability

Data are available on reasonable request. The dataset used to conduct the analyses is available from the corresponding author on reasonable request.
